# No Effect of Cigarette Smoking in the Outcome of Arthroscopic Management for Femoroacetabular Impingement: A Systematic Review

**DOI:** 10.3390/jcm13237214

**Published:** 2024-11-27

**Authors:** Ludovico Lucenti, Nicola Maffulli, Tommaso Bardazzi, Gennaro Pipino, Gaetano Pappalardo, Filippo Migliorini

**Affiliations:** 1 Department of Precision Medicine in Medical, Surgical and Critical Care (Me.Pre.C.C.), University of Palermo, 90133 Palermo, Italy; 2 Department of Medicine and Psychology, University of Rome “La Sapienza”, 00185 Rome, Italy; 3 Centre for Sports and Exercise Medicine, Barts and the London School of Medicine and Dentistry, Mile End Hospital, Queen Mary University of London, London E1 4DG, UK; 4 School of Pharmacy and Bioengineering, Keele University Faculty of Medicine, Stoke on Trent ST4 7QB, UK; 5 Department of Orthopaedic and Trauma Surgery, Academic Hospital of Bolzano (SABES-ASDAA), 39100 Bolzano, Italymigliorini.md@gmail.com (F.M.); 6 Department of Orthopedics and Trauma Surgery, Villa Erbosa Hospital, San Raffaele University of Milan, 20132 Milano, Italy; 7 Department of Orthopedic Surgery, Oberlinklinik GmbH, 14482 Potsdam, Germany; 8 Department of Life Sciences, Health, and Health Professions, Link Campus University, 00165 Rome, Italy

**Keywords:** femoroacetabular impingement, FAI, smoking, cigarettes

## Abstract

**Background:** The impact of smoking in arthroscopic surgery for femoroacetabolar impingement (FAI) is controversial. This systematic review updates and discusses current evidence on the influence of cigarette smoking on the outcome of arthroscopic management of FAI. The outcomes of interest were to compare patient-reported outcome measures (PROMs) and complications. **Methods:** The present systematic review followed the PRISMA guidelines. Embase, Web of Science, and PubMed were accessed in June 2024 without additional filters or temporal constraints. All the clinical investigations comparing smokers versus nonsmokers in patients who underwent arthroscopic management for FAI were considered. The risk of bias in nonrandomised controlled trials was assessed using the Risk of Bias in Nonrandomised Studies of Interventions (ROBINS-I). **Results:** Data from 368 patients were retrieved. The mean length of follow-up was 34.1 ± 7.1 months. The mean age was 40.4 ± 4.0 years and the mean BMI was 27.1 ± 1.6 kg/m^2^. No significant difference was evidenced in the visual analogue scale, Harris hip score, Hip Outcome Score—Sport subscale, and Non-Arthritic Hip Score. No difference was observed in the complication rate: reoperation (*p* = 0.6) and progression to THA (*p* = 0.4). **Conclusions:** Tobacco smoking does not appear to influence the outcomes of arthroscopic management for FAI. At approximately 34 months of follow-up, no difference was found in pain, PROMs, reoperation rate, and progression to THA.

## 1. Introduction

Femoroacetabular impingement (FAI) is common [1,2,3] and is the primary cause of pain and tenderness in young and active patients without hip dysplasia [4,5,6,7]. The first scientific mention of hip impingement dates back to 1936 [8]. In 2001, Ganz et al. [9] first described the pathoanatomical deformities of FAI: convexity at the femoral head–neck confluence (cam morphology), over-coverage of the acetabulum rim (pincer morphology), or their combination (mixed morphology) [10,11,12,13,14,15,16,17,18,19,20,21,22,23,24,25,26,27,28,29,30,31]. If left untreated, FAI causes labral and chondral lesions [32,33], possibly associated with early-onset osteoarthritis [5,6,34,35,36]. FAI damages the function and efficacy of the hip, and athletes need to modify their intensity and activity or, in advanced cases, change or retire from activity [3,37,38]. Plain radiographs help detect FAI and evaluate its severity and progression [39]. Magnetic resonance imaging primarily assesses chondral and labral lesions [40,41,42,43]. Computer tomography is appropriate for surgical planning in challenging cases [44,45,46,47,48,49,50,51,52,53]. Several options for managing FAI have been described, involving conservative and surgical options [54,55]. However, clear recommendations are missing [2,55]. Nonsurgical management for FAI focuses on reducing symptoms, improving joint function, and preventing damage progression to the hip joint [56]. Nonsurgical management consists of physical therapy, modification of the activity of daily living, nonsteroidal anti-inflammatory drugs (NSAIDs), corticosteroid injections, manual therapy, weight management, and education [57,58]. However, in selected patients, surgical intervention may be considered. Arthroscopy is effective for FAI [3,25,59,60,61,62,63], with superior results to conservative approaches [7,62,64]. Despite these generally positive outcomes, the results of arthroscopy strictly depend on proper patient selection and characteristics [65,66,67,68]. Therefore, various demographic, anatomical, diagnostic, habit, and therapeutic prognostic factors must be evaluated. Among them, cigarette smoking has attracted attention. Tobacco is among the most studied risk factors in several medical disciplines [69,70,71,72,73,74,75,76,77,78]. Smoking prevalence in younger people is high, and the worldwide number of cigarette smokers will reach 1.7 billion by 2025 [79,80]. The impact of smoking on the outcome of surgical procedures in different surgical disciplines is well documented [81,82]. Smoking reduces physical performance, wound healing, and mobility. In FAI, evidence of the effect of tobacco on post-surgical outcomes is limited. Only a few comparative studies have been published evaluating the influence of smoking [83,84,85], and a systematic review is missing. Therefore, this study compared the outcomes of smokers versus nonsmokers treated arthroscopically for FAI. The present investigation systematically updates and discusses current evidence on the influence of cigarette smoking on the outcome of arthroscopic management of FAI. The outcomes of interest were to compare patient-reported outcome measures (PROMs) [86,87,88,89,90] and complications.

## 2. Methods

### 2.1. Eligibility Criteria

All clinical studies on arthroscopic management of FAI were considered. Only studies that compared a population of smokers and a population of nonsmokers who underwent comparable procedures were included. According to the authors’ capabilities, only articles published in the following languages were included: English, Spanish, Italian, French, or German. Only studies published in peer-reviewed journals and classified as levels I to III of evidence, according to the 2020 Oxford Centre of Evidence-Based Medicine [91], were included. Reviews, letters, editorials, and opinions were excluded. Studies involving in vitro or animal experiments, cadaveric research, computational analyses, or biomechanical assessments were also disregarded. Finally, only studies with a minimum of 24 months of follow-up were considered.

### 2.2. Search Strategy

The present systematic review followed the guidelines defined in the 2020 Preferred Reporting Items for Systematic Reviews and Meta-Analyses (PRISMA) [92] and the recommendations of the Cochrane Handbook for Systematic Reviews of Interventions [93]. The literature search followed the PICOTD algorithm:

P (Problem): FAI;
I (Intervention): arthroscopy;
C (Comparison): smokers vs. nonsmokers;
O (Outcomes): complications and PROMs;
T (Timings): minimum 24 months of follow-up;
D (Design): clinical study.

Embase, Web of Science, and PubMed were accessed on 27 June 2024, without additional filters or temporal constraints. Other databases were not considered. The following Medical Subject Headings (MeSH) were used: (“Acetabulum”[Mesh] OR “Acetabulum/injuries”[Mesh] OR “Acetabulum/pathology”[Mesh] OR “Cartilage”[Mesh] OR “Cartilage/physiopathology”[Mesh] OR “Femoracetabular Impingement”[Mesh] OR “Femoracetabular Impingement/epidemiology”[Mesh] OR “Femoracetabular Impingement/pathology”[Mesh] OR “Femoracetabular Impingement/physiopathology”[Mesh] OR “Fibrocartilage/physiopathology”[Mesh] OR “Hip”[Mesh] OR “Hip Joint/pathology”[Mesh] OR “Pain”[Mesh] OR Acetabulum OR cam OR Cam impingement OR cam lesion OR cartilage OR chondral lesion OR Conflict OR FAI OR FAI syndrome OR Femoracetabular impingement OR Femoro-acetabular impingement OR impingement OR Pincer impingement) AND (“Acetabulum/surgery”[Mesh] OR “Arthroscopy”[Mesh] OR “Arthroscopy/methods”[Mesh] OR “Arthroscopy/standards”[Mesh] OR “Debridement”[Mesh] OR “Femoracetabular Impingement/surgery”[Mesh] OR “Fibrocartilage/surgery”[Mesh] OR “Hip Joint/surgery”[Mesh] OR acetabular labral refixation OR Acetabular Rim Resection OR arthroscopic labral reconstruction OR arthroscopic surgery OR Arthroscopy OR Debridement OR hip arthroscopic surgery OR Hip arthroscopy OR labral reconstruction OR labral repair OR labrum repair OR Reconstruction OR Repair) AND (“Smokers”[Mesh] OR “Ex-Smokers”[Mesh] OR “Non-Smokers”[Mesh] OR Smokers OR Smoking history OR ex smokers OR non smokers) AND (“Patient Outcome Assessment”[Mesh] OR “Patient Reported Outcome Measures”[Mesh] OR “Patient Satisfaction”[Mesh] OR “Quality of Life”[Mesh] OR “Visual Analog Scale”[Mesh] OR clinical outcome OR Harris Hip Score OR hip outcome score OR patient outcomes OR Patient Reported Outcome Measures OR Treatment outcome OR VAS OR visual analog scale).

### 2.3. Selection and Data Collection

Two authors (G.P. and L.L.) independently conducted the database search. All the titles underwent manual screening and their abstracts were reviewed if deemed relevant. The full texts of articles matching the inclusion criteria were scrutinised. Articles lacking accessible full texts were excluded. Furthermore, the bibliographies of full-text articles were cross-referenced for potential inclusion. Any discrepancies between authors were resolved by a third author (N.M.), who made the final decision.

### 2.4. Data Items

Two authors (T.B. and L.L.) independently extracted and collected data using Microsoft Office Excel version 16.0 (Microsoft Corporation, Redmond, WA, USA). The following generalities were collected for each study: author, year of publication, journal, study design, and length of follow-up. The following data at baseline were extracted: number of patients, women, and BMI. Data concerning the visual analogue scale (VAS) [94], modified Harris Hip Score (mHHS) [95], Hip Outcome Score—Sport-Specific Subscale (HOS-SSS) [96], and Non-Arthritic Hip Score (NAHS) [97] were collected at baseline and last follow-up. Data concerning the following complications were retrieved: revision and progression to THA.

### 2.5. Assessment of the Risk of Bias

The risk of bias evaluation adhered to the rigorous guidelines outlined in the Cochrane Handbook for Systematic Reviews of Interventions, ensuring a systematic and transparent assessment of study quality [93]. This evaluation is critical for minimising systematic errors that could compromise the validity of a systematic review’s findings. Two authors (G.P. and F.M.) independently assessed the risk of bias in the included studies to enhance objectivity and reliability. Disagreements, if any, were resolved through discussion or consultation with a third reviewer to achieve consensus.

The Risk of Bias in Nonrandomised Studies of Interventions (ROBINS-I) tool was used [98] for nonrandomised controlled trials. This comprehensive tool evaluates the risk of bias across seven domains, addressing distinct stages of the study design, implementation, and reporting. These domains include confounding factors and patient selection characteristics before the comparative intervention, bias in classification during the intervention, methodological quality postintervention comparison, which involves deviations from intended interventions, missing data, inaccurate outcome measurement, and bias in reported outcome selection. The application of ROBINS-I provides a nuanced categorisation of the risk of bias for each study as “Low”, “Moderate”, “Serious”, or “Critical”, depending on the extent and nature of the identified biases. Each domain was carefully examined, and judgments were made based on predefined criteria to ensure consistency. To visually summarise the risk of bias assessments, a chart was generated using the Robvis software (Risk-of-bias VISualization, Riskofbias.info, Bristol, UK, https://mcguinlu.shinyapps.io/robvis/) [99]. This tool facilitates the creation of transparent and interpretable graphics, such as weighted bar charts or traffic-light plots, which provide an at-a-glance overview of the methodological quality of the included studies.

### 2.6. Synthesis Methods

The main author (F.M.) performed the statistical analyses. Descriptive statistics were calculated using the IBM SPSS software version 25 (International Business Machines Corporation, Armonk, NY, USA). The arithmetic mean and standard deviation were used for continuous data and the frequency (events/observations) for dichotomic variables.

## 3. Results

### 3.1. Study Selection

**Figure 1:**
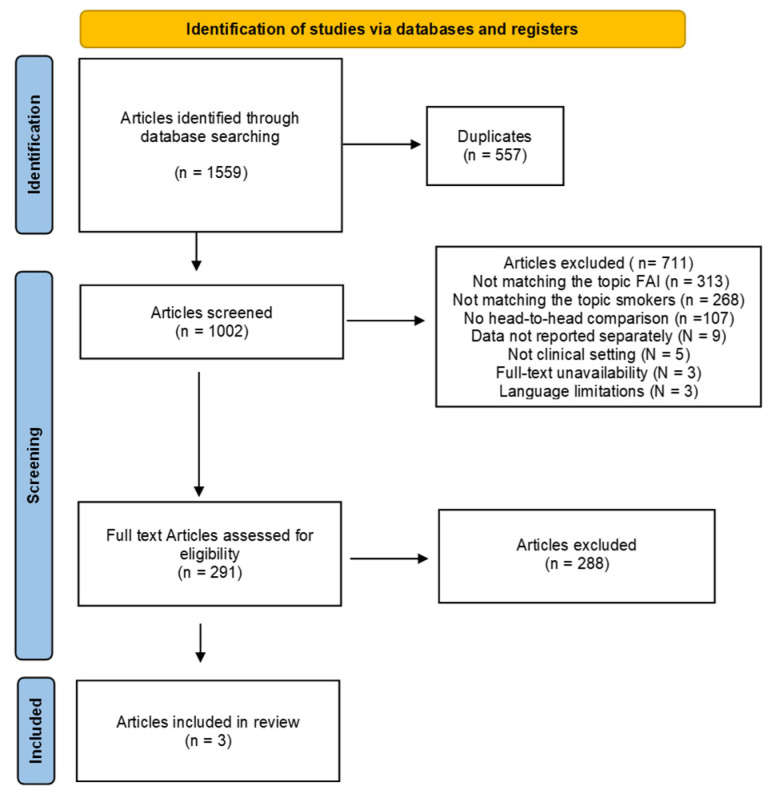
PRISMA flow chart of the literature search.

The systematic literature search resulted in the identification of 1559 articles. After removing duplicates, the abstracts of 1002 articles were screened for eligibility. A total of 711 articles were excluded for the following reasons: not matching the topic FAI (N = 313), not matching the topic smokers (N = 268), no head-to-head comparison (N = 107), data not reported separately (N = 9), not a clinical setting (N = 5), full-text unavailability (N = 3), and language limitations (N = 3). Finally, three studies were included in this systematic review. The results of the literature search are shown in Figure 1.

### 3.2. Risk of Bias Assessment

**Figure 2:**
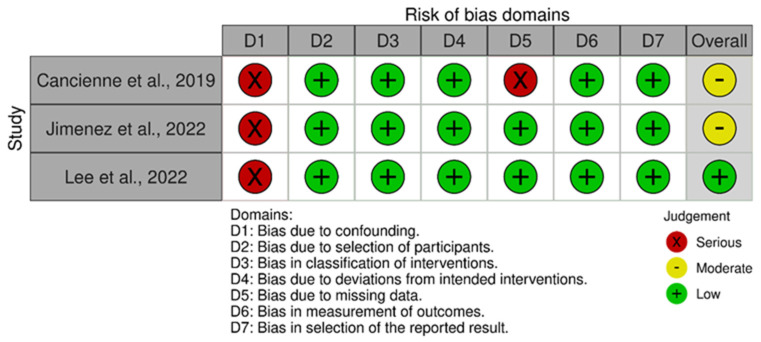
Cochrane risk of bias tool graph [83,84,85].

The ROBINS-I tool was employed to comprehensively evaluate the risk of bias in the selected nonrandomised controlled trials, which comprised three articles. This tool facilitates a domain-specific analysis to identify potential methodological weaknesses that could compromise the validity of study findings. A critical issue was observed in the first domain, as all studies demonstrated a serious risk of bias due to confounding factors. This represents a major limitation, as confounding can significantly impact the comparability of study groups, potentially leading to distorted estimates of intervention effects. Examples of confounding factors could include baseline differences in participant characteristics, such as age, comorbidities, or socioeconomic status, which were either insufficiently accounted for or inadequately adjusted in the analyses. In contrast, the risk of bias arising from participant selection and classification of interventions was consistently low across all non-RCTs. This indicates that the studies implemented appropriate methods to assign participants to intervention groups and accurately categorised the interventions under investigation. Moreover, no deviations from the intended intervention protocols were detected, suggesting the implementation fidelity was well maintained. However, concerns were noted in the domain of missing data for one of the studies. Missing data can compromise the internal validity of a study, especially if the reasons for missing data are related to the intervention or outcome. The domains related to outcome measurement and selection of reported results were deemed as having low risk of bias across all articles. This indicates that the outcome assessments were conducted using valid and reliable methods, likely with appropriate blinding, and that the reported results were complete and aligned with the prespecified objectives, minimising the risk of selective reporting. Overall, the ROBINS-I assessment revealed a moderate overall risk of bias in two articles. At the same time, the remaining non-RCT was judged to have a low overall risk of bias, suggesting a generally acceptable level of methodological quality. However, the serious confounding bias observed in all studies remains a notable limitation that readers should consider when interpreting the findings (Figure 2).

### 3.3. Study Characteristics and Results of Individual Studies

Data from 368 patients were retrieved. The mean length of follow-up was 34.1 ± 7.1 months. The mean age was 40.4 ± 4.0 years and the mean BMI was 27.1 ± 1.6 kg/m^2^. The generalities of the included studies are shown in Table 1.

### 3.4. Baseline Comparability

The mean length of follow-up, mean age, mean BMI, female/male ratio, VAS, mHHS, HOS-SSS, and NAHS were comparable between the two groups (Table 2).

### 3.5. Synthesis of Results

No significant difference was evidenced in VAS, mHHS, HOS-SSS, and NAHS (Table 3).

No difference was observed in the rate of complication (Table 4): reoperation (*p* = 0.6) and progression to THA (*p* = 0.4).

## 4. Discussion

Femoroacetabular impingement (FAI) is a chronic hip condition caused by mechanical conflict in the femoral head–neck junction and/or an acetabular over-coverage, which leads to soft tissue damage of the hip joint [25,38,100]. Other modifiable factors impacting FAI include physical activity level, body mass index (BMI), muscle strength and flexibility, and posture and movement patterns [101,102,103,104]. Over time, labral and cartilage injury might lead to hip osteoarthritis and persistent pain. The diagnosis of FAI consists of the patient’s anamnesis and physical examination. Radiography of the hip and pelvis is also necessary to pose a definite diagnosis. Initially, the initial management includes nonoperative modalities. If conservative therapy fails, surgical management could be considered [105]. Hip arthroscopy effectively manages symptomatic FAI in patients with no signs of advanced osteoarthritis [61,106,107]. The prevalence of FAI in the adult population is between 10% and 15%, with greater prevalence in young athletes [5,108,109,110]. Understanding modifiable risk factors, such as tobacco smoking, is critical for prevention and management of this condition. In the current literature, the influence of cigarette smoking on surgical outcomes has been investigated [83,111,112]. Carbon monoxide induces a hypoxic environment, which, combined with the platelet-activating and vasoconstrictive effects of nicotine, impairs healing [113]. Intracellular collagen synthesis and fibroblast and macrophage activity are hindered by tobacco smoking [114,115]. Therefore, tobacco smoking could lead to detrimental effects on bone, labral, and capsular healing after hip arthroscopy for FAI [114,116]. The present systematic review discusses the current evidence of tobacco smoking on the outcome of patients who have undergone arthroscopy for FAI.

According to the main findings of the present systematic review, tobacco smoking does not impair the outcomes of arthroscopy for FAI. At approximately 34 months of follow-up, no difference was found in pain, PROMs, reoperation rate, and progression to THA. The effect of smoking on musculoskeletal pathologies and surgical outcomes is documented in the current literature [117,118,119,120,121,122]. Smokers experience poorer outcomes after joint arthroplasty and vertebral surgery [123,124,125]. Although arthroscopic procedures have a low risk of postoperative complications [7,126,127,128], Heyer et al. [121] found that smoking is an independent risk factor for complications in several arthroscopic procedures in the knee and shoulder. Emara et al. [129], analysing 18,585 hip arthroscopies, stated that smoking negatively impacts PROMs and is associated with a higher rate of postoperative complications. The present systematic review found no statistically significant differences in pain between smokers and nonsmokers. This contrasts with some studies that indicate smoking is correlated with increased pain perception and poorer pain management outcomes [130,131]. Some studies demonstrated an association between smoking and musculoskeletal pain [132,133,134,135,136]. On the other hand, some authors stated that smokers with pain were less likely to initiate a smoking cessation attempt compared to smokers with no pain [137]. The absence of substantial differences in pain in the present study suggests that other factors may be involved in determining pain outcomes.

Regarding the functional scores investigated (mHHS, NAHS, and HOS-SSS), data showed no differences between smokers and nonsmokers. This is coherent with previous studies, which found that smoking does not substantially impact functional recovery succeeding orthopaedic surgeries [138]. The impact of smoking on perioperative and postoperative outcomes varies depending on the surgical procedure [139]. However, other research points to the adverse effects of smoking on postoperative recovery and long-term functional outcomes (other than complications, inpatient mortality, and persistent opioid consumption) [140]. The present systematic review showed a comparable rate of reoperation and progression to THA between smokers and nonsmokers. This contrasts previous evidence, which reported a higher complication rate among smokers, particularly regarding wound healing and infection [141,142]. A recent systematic review [142] emphasised that former smokers had fewer complications when compared to active smokers. The disagreement in the current data could result from the specific patient population, the low sample, or the different types of surgery. The present systematic review has several limitations. The examined studies only considered arthroscopy for FAI [143,144], while other types of surgery, such as open dislocation or mini-open procedures [145,146,147], were excluded. A possible association between smoking habits and the type of surgery was not investigated. Furthermore, there was considerable heterogeneity about several key elements that might affect the results. Arthroscopy for FAI varies with the kind of pathology and existing damage [54], it includes addressing the cam/pincer deformity, osteoplasty, and the re-establishment, repair, or reconstruction of the acetabular labrum or the cartilage [54]. In the analysed studies [83,84,85], differences in the surgical technique were not considered. Similarly, the patient selection, surgical indications, and other primary factors, such as the rehabilitation protocol, were not evaluated. Therefore, these findings are challenging to generalise. Given the heterogeneous and limited data available for inclusion, conducting a meta-analysis was not possible. The restricted sample sizes, the limited number of articles, and the lack of standardised protocols across the studies impair data synthesis. Only one [83] investigation was prospective, while the other two were retrospective [84,85]. All the studies reported that they were conducted in one institution, and all the patients were operated on only by one surgeon in a standardised fashion. Smoking status was recorded qualitatively: it was considered a binary yes/no variable, but the exact number of cigarettes per day or year was not provided. Patient smoking status was assessed preoperatively, but it was not re-evaluated at the final follow-up; therefore, patients considered within the smoking group may have quit after surgery, while those in the never-smoking cohort may have started smoking at some point.

The association between smoking and lower functional outcome scores is likely multifactorial and may be influenced by the generally poorer health of smokers and other pathologies. Other factors frequently associated with smoking, such as alcohol use and stress levels, were not considered, and this could limit the external validity of these results. No study examined the difference in return to sports. A recent systematic review [38] reported that arthroscopy for FAI resulted in excellent results in terms of return to sport; according to the authors, a total of 88.75% (581 of 655) of patients were able to return to sports within a mean of 37.4 (±16.5 months). Other authors reported similar results [148]. Although results from the present study showed no differences between smokers and nonsmokers, it is necessary to compare them with the existing literature, which suggests that smoking generally causes a higher risk for adverse outcomes. Other factors, such as patient characteristics and the type of intervention, might be additional critical aspects that influence the results. Similarly, information on postoperative rehabilitation modalities is often biased and might represent another critical limitation. Several significant gaps remain in the literature that underscore the importance of this study. Despite known associations between smoking and musculoskeletal conditions, few studies specifically investigate smoking as a potential risk factor for FAI. Most existing research focuses on factors like genetics, biomechanics, or physical activity, with smoking often overlooked as a contributing factor. An inter-rater reliability assessment was not conducted during the search, which might reduce the validity of the present study. Given the lack of quantitative data and missing information, additional subgroup analyses according to the average amount of nicotine and cigarettes smoked daily were not possible. To increase the applicability of these findings on a larger scale, future studies should use better methodological controls and include larger sample sizes to enhance validity. Furthermore, future research should aim to quantify smoking habits more accurately to identify any threshold levels of smoking in the perioperative period that led to the poorer outcomes observed.

## 5. Conclusions

Tobacco smoking does not influence the outcomes of arthroscopic management for FAI. At approximately 34 months of follow-up, no difference was found in pain, PROMs, reoperation rate, and progression to THA.

## Figures and Tables

**Table 1:** Generalities of the included studies.

Author and Year	Journal	Design	Follow-Up (Months)	Smokers	Patients (n)	Women (n)	Mean Age (y)	Mean BMI
Cancienne et al., 2019 [83]	*Am J Sports Med*	Retrospective	24.0	Yes	40	24	35.5	27.0
				No	80	49	36.1	24.6
Jimenez et al., 2022 [84]	*Orthop J Sports Med*	Retrospective	39.9	Yes	20		41.4	30.2
				No	60		42.5	28.7
Lee et al., 2022 [85]	*Orthop J Sports Med*	Retrospective	38.6	Yes	84	62	45.0	26.6
				No	84	58		28.0

**Table 2:** Baseline comparability (VAS: Visual Analogue Scale, mHHS: modified Harris Hip Score, NAHS: Non-Arthritic Hip Score, HOS-SSS: Hip Outcome Score—Sport-Specific Subscale).

Endpoint	Smokers (N = 144)	Nonsmokers (N = 224)	*p*
Women	69.4% (86 of 124)	65.2% (107 of 164)	0.8
Mean follow-up (*months*)	34.7 ± 6.7	33.7 ± 7.3	0.5
Age (*mean*)	41.9 ± 4.1	38.8 ± 3.2	0.4
BMI (*mean*)	27.2 ± 1.2	27.0 ± 1.8	0.7
VAS (*mean*)	6.2 ± 2.2	6.0 ± 2.2	0.7
mHHS (*mean*)	58.2 ± 13.4	61.3 ± 15.3	0.2
NAHS (*mean*)	57.7 ± 16.6	60.2 ± 15.6	0.5
HOS-SSS (*mean*)	42.2 ± 21.9	47.8 ± 22.7	0.9

**Table 3:** Results of PROMs (VAS: visual analogue scale, mHHS: modified Harris Hip Score, NAHS: Non Arthritic Hip Score, HOS-SSS: Hip Outcome Score—Sport-Specific Subscale).

Endpoint	Smokers (N = 144)	Nonsmokers (N = 224)	Effect Size	*p*
VAS (*mean*)	2.3 ± 2.7	1.9 ± 2.4	0.4	0.4
mHHS (*mean*)	82.6 ± 20.6	84.1 ± 15.5	−1.5	0.6
NAHS (*mean*)	85.7 ± 13.5	85.4 ± 17.8	0.3	0.8
HOS-SSS (*mean*)	65.7 ± 17.1	69.8 ± 14.1	−4.1	0.6

**Table 4:** Results of the outcome: complications.

Endpoint	Smokers (N = 144)	Nonsmokers (N = 224)	*p*
Reoperation	5.8% (6 of 104)	4.9% (7 of 144)	0.6
Progression to THA	7.7% (8 of 104)	2.8% (4 of 144)	0.4

## Data Availability

The datasets generated during and/or analysed during the current study are available throughout the manuscript.
